# Glucose and acute exercise influence factors secreted by circulating angiogenic cells in vitro

**DOI:** 10.14814/phy2.12649

**Published:** 2016-02-04

**Authors:** Sarah Witkowski, Gayatri Guhanarayan, Rachel Burgess

**Affiliations:** ^1^ Department of KinesiologyUniversity of Massachusetts AmherstAmherstMassachusetts

**Keywords:** Acute exercise, circulating angiogenic cells, cytokine, diabetes, inflammation, nitric oxide

## Abstract

Circulating angiogenic cells (CAC) influence vascular repair through the secretion of proangiogenic factors and cytokines. While CAC are deficient in patients with diabetes and exercise has a beneficial effect on CACs, the impact of these factors on paracrine secretion from CAC is unknown. We aimed to determine whether the in vitro secretion of selected cytokines and nitric oxide (NO) from CAC is influenced by hyperglycemia and acute exercise. Colony‐forming unit CAC (CFU‐CAC) were cultured from young active men (*n* = 9, 24 ± 2 years) at rest and after exercise under normal (5 mmol/L) and elevated (15 mmol/L) glucose. Preliminary relative multiplex cytokine analysis revealed that CAC conditioned culture media contained three of six measured cytokines: transforming growth factor‐beta‐1 (TGF *β*1), tumor necrosis factor alpha (TNF *α*), and monocyte chemotactic protein‐1 (MCP‐1). Single quantitative cytokine analysis was used to determine the concentration of each cytokine from the four conditions. NO was measured via Griess assay. There was a significant effect of CAC exposure to in vivo exercise on in vitro TGF *β*1 secretion (*P* = 0.024) that was independent of glucose concentration. There was no effect of glucose or acute exercise on TNF *α* or MCP‐1 concentration (both *P* > 0.05). The concentration of NO from CFU‐CAC cultured in elevated glucose was lower following acute exercise (*P* = 0.002) suggesting that exercise did not maintain NO secretion under hyperglycemic conditions. Our results identify paracrine signaling factors that may be responsible for the proangiogenic function of CFU‐CAC and an influence of acute exercise and elevated glucose on CFU‐CAC soluble factor secretion.

## Introduction

Vascular regenerative cell types including circulating angiogenic cells (CAC) support vessel remodeling via differentiation into endothelial cells and/or the secretion of paracrine factors (Sahoo et al. 2011; Hynes et al. 2013; Landers‐Ramos et al. 2015) and have been identified via cell surface markers (e.g., CD34, VEGFR2, CD133) and characteristics (e.g., colony formation and acetylated LDL uptake) (Rehman et al. 2003; Urbich et al. 2005). One type of CACs, colony‐forming CAC or CFU‐CAC, has been defined as “early outgrowth” endothelial progenitors derived from the peripheral mononuclear fraction. Colony‐forming CAC are now recognized as a mixed cell population that consist largely of hematopoietic cells including monocytes and lymphocytes in addition to CD34^+^ endothelial progenitors (Rohde et al. 2006; Yoder et al. 2007). CFU‐CAC are related to endothelial function measured via flow‐mediated brachial reactivity and conventional cardiovascular disease (CVD) risk factors (Hill et al. 2003). One explanation for this relationship is that the complement of cells that comprise CFU‐CAC secrete factors to influence endothelial cell function (Rohde et al. 2006, 2007; Pula et al. 2009). Cytokines regulate innate and adaptive immune responses and proinflammatory cytokines are recognized for their role in endothelial dysfunction (Aroor et al. 2013; Shao et al. 2014). Therefore, cell‐derived factors, including cytokines and chemokines, may be important for blood vessel homeostasis and evaluation of variables that influence their release and action can improve our understanding of vascular function in a variety of diseases and conditions.

Patients with type 2 diabetes (T2DM) are at higher risk for CVD and experience greater rates of macro‐ and microvascular complications than nondiabetics (Bernardi et al. 2012) due in part to the effect of hyperglycemia on the endothelium. Endothelial cells exposed to hyperglycemic conditions have increased inflammatory gene expression (Piconi et al. 2004; Piga et al. 2007) and decreased nitric oxide (NO) production (Ding et al. 2000; Du et al. 2001). Many CAC populations, including early endothelial progenitors, have been shown to be lower in number and function in patients with diabetes compared with controls (Tepper et al. 2002) and negatively related to fasting plasma glucose level (Aschbacher et al. 2012). CFU‐CAC formation and skeletal muscle capillarization was reported to be lower in adults with impaired glucose tolerance compared with nondiabetic control subjects and CFU‐CAC number was directly associated with capillary density (Prior and Ryan 2013). Nitric oxide signaling is strongly related to CAC function, and appears to be deficient in CAC from patients with diabetes and when CAC are exposed to superphysiological glucose levels (Chen et al. 2007; Cubbon et al. 2010; Heiss et al. 2010). Alteration of the amount and type of factors secreted from CFU‐CAC in different glycemic environments may influence the relationship between CFU‐CAC and the vasculature.

Exercise is well recognized for its benefits on cardiovascular health, diabetes, and blood vessel growth and repair. Acute exercise stimulates glucose uptake into skeletal muscle and exercise training has important insulin‐sensitizing and metabolic effects that improve regulation of blood glucose. Similarly, acute bouts of exercise, exercise training, and reduced physical activity can influence CAC. Specifically, as little as one acute bout of exercise increases, whereas 10 days of reduced physical activity decreases the number of CFU‐CAC from healthy active young men (Jenkins et al. 2009; Guhanarayan et al. 2014). Furthermore, acute exercise influences gene expression and increases intracellular nitric oxide (NOi) within cells cultured in CAC colonies (Jenkins et al. 2009). However, there is limited available evidence on effect of exercise on the secretion of factors from CAC that influence vascular growth and endothelial function (Landers‐Ramos et al. 2015). Therefore, the purpose of this study was to evaluate whether elevated glucose levels similar to those found in patients with poorly controlled diabetes, would influence the release of NO and cytokine signaling factors from CFU‐CAC in vitro and whether an acute bout of exercise altered this response. We hypothesized that CAC cultured in elevated glucose would exhibit lower secretion of NO and growth‐related cytokines and greater release of proinflammatory cytokines compared with normal glucose. We also hypothesized that an acute bout of exercise would normalize the glucose effect on secreted factors from CFU‐CAC.

## Materials and Methods

### Subjects

All protocols were approved by the University of Massachusetts Institutional Review Board. Written informed consent was obtained from all participants. Healthy, nonsmoking, medication‐free males (*N* = 9, 24 ± 2 years) with no history of CVD or diabetes were recruited for this study. Participants were screened using health history and physical activity questionnaires and were included if they engaged in at least 150 min/week of moderate to vigorous physical activity including running to eliminate responses to the treadmill exercise due to lack of familiarity with that mode of exercise during testing. Resting heart rate and blood pressure were also measured during the screening visit.

### Exercise and blood sampling

All testing was performed in the morning following an 8‐h overnight fast. Participants refrained from regular exercise for 48 h prior to testing to eliminate the effect of acute exercise. Fourteen ml of blood was drawn into tubes that contained the anticoagulant ethylenediamine tretraacetic acid before and after an acute bout of moderate treadmill exercise. Participants rested in the supine position for 30 min prior to the preexercise blood draw. Following cool‐down, participants returned to supine rest for 15 min prior to the postexercise blood draw. Exercise consisted of running for 30 min at 70% of heart rate reserve (HRRmax) with an additional 3‐min warm‐up and cool down. HRRmax was calculated using the Karvonen method and age‐predicted maximum heart rate. Continuous heart rate monitoring and rating of perceived exertion (RPE, Borg scale) were measured to monitor the participants during exercise to ensure that they remained within the prescribed intensity.

### CFU‐CAC isolation and culture

Peripheral blood mononuclear cells (PBMC) were isolated from blood drawn before and after exercise via Ficoll density gradient centrifugation and cultured using the assay developed by Hill et al. (2003) with minor modifications. Briefly, 5 × 10^6^ PBMC per well were plated on a fibronectin plate for 48 h in either normal glucose media (5 mmol/L, equivalent to 90 mg/dL) or high glucose media (15 mmol/L, equivalent to 270 mg/dL). Nonadherent cells were collected, counted and replated at a density of 1 × 10^6^ cells per well and cultured in either normal or high glucose media for an additional 3 days. On the 5th day, colony‐forming units (CFU) were counted and culture media were collected and frozen at −80°C from each condition (preexercise/normal glucose, preexercise/high glucose, postexercise/normal glucose, and postexercise/high glucose) until analysis. Colony‐forming units were enumerated for each condition. A CFU was defined as a central core of round cells with sprouting elongated cells around the periphery. The endothelial lineage of these cells has been previously confirmed via staining for CD31 and VEGFR2 (Hill et al. 2003). CFU‐CAC and have also been shown to express T‐cell and monocyte markers (Rohde et al. 2006, 2007; Medina et al. 2010).

### Cytokine secretion analysis

To determine whether the selected cytokines were secreted from CACs, the relative amount of six candidate cytokines from CAC conditioned media under resting (nonexercised, *n* = 2) conditions was measured using a custom Multi‐analyte ELISArray assay (Qiagen, Germantown, MD) as per instructions from the manufacturer. Candidate cytokines were chosen according to their relationship with vascular inflammation, CAC signaling, and diabetes (Schober 2008; Yang et al. 2009; Zhang et al. 2009; Chen et al. 2011; Desouza et al. 2011; Rissanen et al. 2012) and included tumor necrosis factor alpha (TNF*α*), granulocyte‐macrophage colony‐stimulating factor (GM‐CSF), granulocyte colony‐stimulating factor (G‐CSF), monocyte chemotactic protein‐1 (MCP‐1), transforming growth factor‐beta‐1 (TGF*β*1), and interleukin 1 beta (IL‐1*β*). Positive controls for each cytokine and control media, in which no cells were cultured, were included in the analysis. To quantify the absolute concentration of cytokines for each condition (glucose concentration and exercise), cell culture supernatants were assayed using the Single Analyte ELISArray (Qiagen). For both assays, cell culture media were thawed and spun at 1000× g to remove debris prior to performing the assay. All cytokines/chemokines were measured in duplicate and ELISA plates were read at an absorbance of 450 nm with a correction absorbance of 570 nm. Total protein content in the media was assayed via Pierce BCA protein assay (Thermo Scientific, Waltham, MA). There was no effect of exercise or glucose on total protein content (*P* > 0.05).

### Nitric oxide assay

Nitrate/nitrite content in the media was measured with the Griess Assay (Cayman, Ann Arbor, MI) via the manufacturer's specifications. Briefly, this assay converts nitrate to nitrite with the enzyme nitrate reductase then uses the Griess Reagents to convert nitrite into the Azo chomophore compound that which can be evaluated through reading the absorbance at 540 nm. Media from the four conditions were thawed on ice and spun at 1000× g to remove debris. The supernatant was assayed in triplicate. Interassay coefficient of variation (CV) was 6.98 ± 0.74% and intra‐assay CV was 8.07 ± 2.69%.

### Gene expression

After 5 days of culture, cells from each of the conditions were washed and RNA isolated via TRI reagent (Sigma‐Aldrich, St. Louis, MO) according to the manufacturer's specifications. RNA was quantified via absorbance at 260/280 nm on a spectrophotometer (NanoDrop; Thermo Scientific, Agawam, MA). One microgram of total RNA was reverse transcribed to cDNA via iScript reverse transcriptase (Bio‐Rad, Hercules, CA) according to the manufacturer's instructions. Gene expression was determined for using real‐time PCR (RT‐PCR, CFX‐96; BioRad) and normalized to *GAPDH* (forward primer, 5′‐ACCCAGAAGACTGTGGATGG‐3′, reverse primer, 5′‐TTCTAG ACGGCAGGTCAGGT‐3′). Transforming growth factor *β*1 (*tgfβ1*) primer sequences were forward, 5′‐CAACACCATGTGGTCTGGAG‐3′ and reverse. 5′‐GCACAGCCCAGT GAGTACAA‐3′. Amplicon characteristics and primer specificity was analyzed via dissociation curves for each assay. Efficiency was >85% for all targets and mRNA expression of the target gene was calculated as 2^−∆CT^ where ∆*C*_T_ is the *C*_T_ of the target gene minus *GAPDH* control for each condition. Fold change in gene expression was calculated relative to the preexercise, normal glucose condition, which was equal to 1.

### Statistical analysis

Data are presented as means ± SEM. Data for each variable were tested to determine that they met the assumptions for statistical testing. Sample size was estimated from a previous analysis on the acute exercise response in CFU‐CAC formation in healthy active young men (Jenkins et al. 2009). According to this analysis, a sample size of 9 would yield 80% power (*α *= 0.05). Two‐way repeated measures ANOVAs were used to evaluate significant interaction (exercise × glucose) effect and main effects of exercise (pre, postexercise), and glucose level (normal, high glucose). Repeated measures ANOVAs were followed by Tukey post hoc testing for individual comparisons when a significant main effect was detected. Pearson correlations were used to evaluate relationships between variables.

## Results

### Subject characteristics

Participants were normotensive and active as they reported over 150 min of moderate to vigorous physical activity per week. Acute exercise yielded a RPE indicative of the targeted moderate intensity exercise (Table 1).

**Table 1:** Participant characteristics

Characteristic	Average ± SEM
Age, year	24 ± 2
Weight, kg	74.7 ± 6.0
Height, m	1.72 ± 0.02
BMI, kg/m^2^	24.9 ± 1.7
SBP, mmHg	118 ± 2
DBP, mmHg	69 ± 2
Physical activity, h/week	7.5 ± 1.2
Resting HR, bpm	52 ± 2
Target HR (70% HRR), bpm	153 ± 2
RPE (Borg scale)	12 ± 1

Data are means ± SEM. SBP, systolic blood pressure; DBP, diastolic blood pressure, RPE, rating of perceived exertion. *N* = 9.

### Cytokines

**Figure 1:**
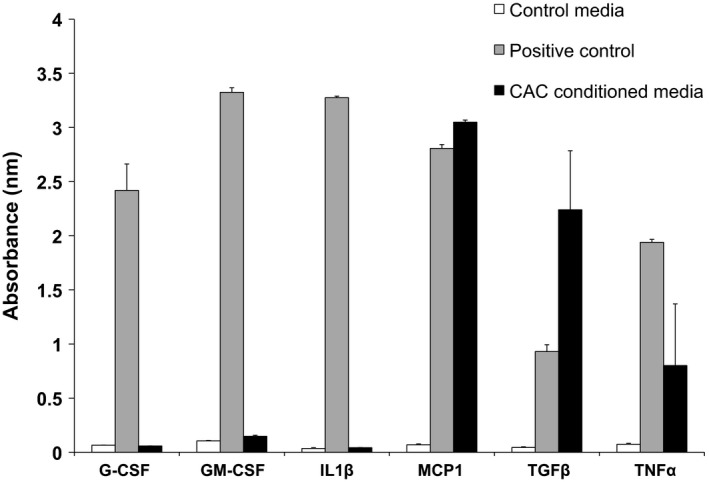
Candidate cytokines released from circulating angiogenic cells. Three of six cytokines were elevated in CAC‐conditioned media under nonexercise, normal glucose (5 mmol/L) conditions relative to control (*N* = 2). CAC, circulating angiogenic cells; G‐CSF, granulocyte colony‐stimulating factor; GM‐CSF, granulocyte‐macrophage colony‐stimulating factor; IL‐1*β*, interleukin‐1 beta; MCP1, monocyte chemotactic protein‐1; TGF *β*, transforming growth factor beta; TNF *α*, tumor necrosis factor alpha. Values are mean ± SEM.

**Figure 2:**
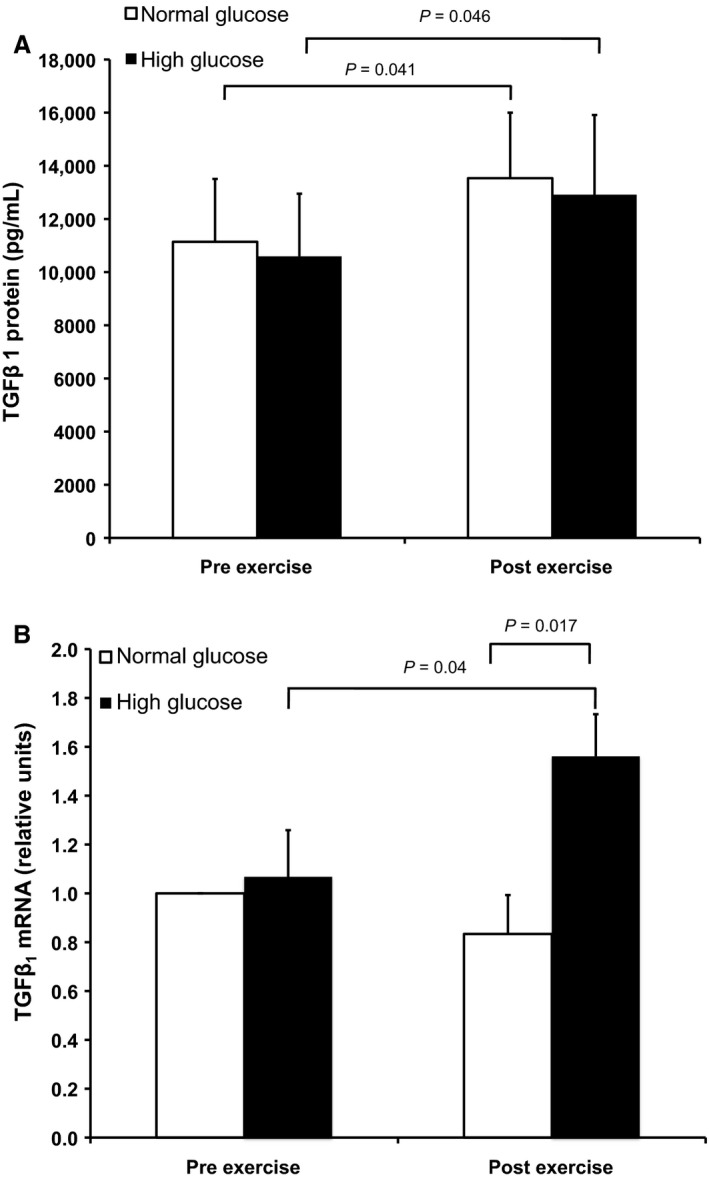
Transforming growth factor *β*1 (TGF *β*1) protein in circulating angiogenic cells (CAC)‐conditioned media was elevated following acute in vivo exercise (A) and CAC gene expression was elevated following acute in vivo exercise after exercise under the high glucose (15 mmol/L, black bars) condition (B). *P*‐values represent post hoc individual comparisons. *N* = 9. Values are mean ± SEM.

Of the six selected cytokines screened via the multiplex assay, TNF*α*, MCP‐1, and TGF*β*1 were detected in CAC‐conditioned media (Fig. 1). Results from the single analyte ELISAs for the four conditions revealed that there was a significant overall main effect of exercise on TGF*β*1 protein in the media (*P* = 0.024) such that TGF*β*1 protein increased with exercise in both normal (Fig. 2A, *P* = 0.041) and high glucose conditions (*P* = 0.046). There was no overall main effect of glucose on TGF*β*1 protein. There was a significant exercise × glucose interaction effect in *Tgfb1* mRNA (*P* = 0.018) such that in the high glucose condition, but not the normal glucose condition, *Tgfb1* mRNA was significantly higher compared with preexercise (Fig. 2B, *P* = 0.04). In CAC cultured after acute exercise, *Tgfb1* mRNA was greater in high versus normal glucose conditions (*P* = 0.017).

**Figure 3:**
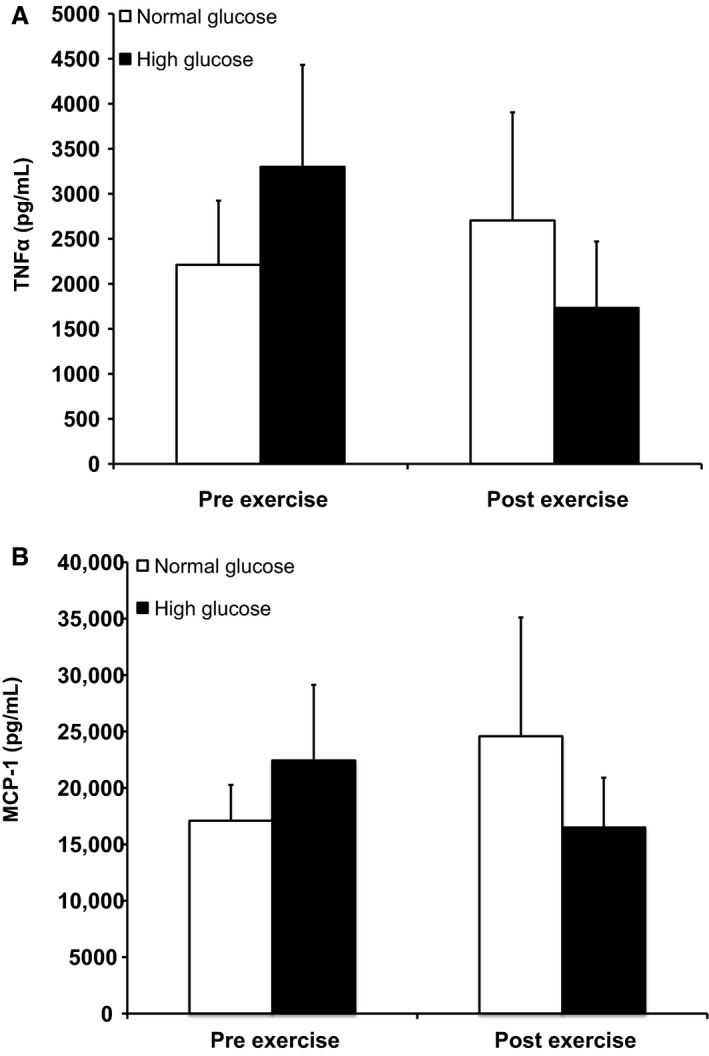
Tumor necrosis factor *α* (A) and monocyte chemotactic protein 1 (B) in circulating angiogenic cells‐conditioned media before and after exercise under normal (5 mmol/L, white bars) and high (15 mmol/L, black bars) glucose conditions. *N* = 9. Values are mean ± SEM.

There was no effect of glucose or exercise on TNF*α* or MCP‐1 secretion. (Fig. 3A, B). However, TNF*α* and MCP‐1 levels were significantly correlated with each other when all conditions were combined (*r* = 0.39, *P* = 0.02). Cytokine levels were not associated with CFU‐CAC number.

### Nitric oxide

**Figure 4:**
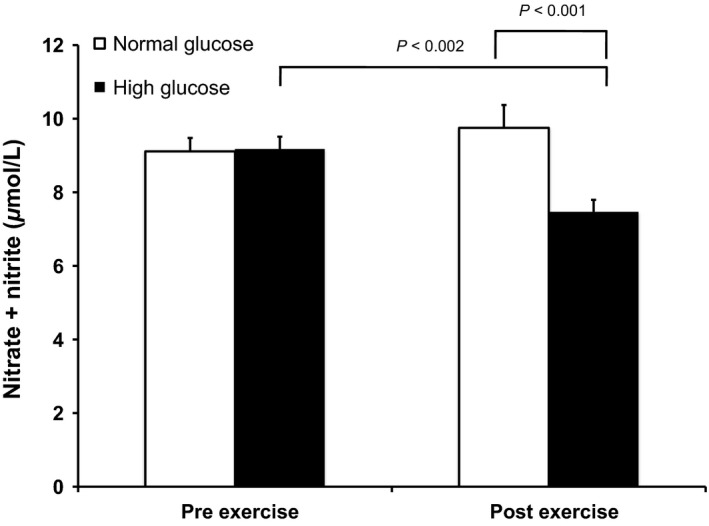
Nitrate/nitrite in circulating angiogenic cells‐conditioned media was significantly decreased after in vivo acute exercise under the high (15 mmol/L, black bars) glucose condition. *P*‐values represent post hoc individual comparisons. *N* = 9. Values are mean ± SE.

There was a significant interaction effect between exercise and glucose level and glucose main effect on nitrate/nitrite appearance in the media (exercise × glucose, *P* = 0.005 and glucose main effect, *P* = 0.031). Acute exercise resulted in lower nitrate/nitrite concentration in the culture media following exercise under high glucose conditions (Fig. 4, *P* < 0.002) such that there was lower nitrate/nitrite detected following exercise in the high glucose compared with normal glucose condition (*P* < 0.001).

### Circulating angiogenic cell colony formation

There was a trend toward high glucose conditions resulting in fewer CAC colonies formed both pre and postexercise (preexercise, normal glucose 16 ± 6 vs. high glucose 10 ± 3 and postexercise, normal glucose 13 ± 5 vs. high glucose 10 ± 3, *P* = 0.066). There was no overall effect of exercise on CAC colony number (*P* > 0.05).

## Discussion

Diabetic conditions induce changes in blood vessel integrity and growth in many tissues. For example, impaired glucose tolerance is associated with reduced skeletal muscle capillary density (Prior et al. 2009). On other hand, diabetic retinopathy is characterized by angiogenic responses following pericyte cell death and vascular lesion development (Sheetz and King 2002). The CAC population evaluated herein is associated with vascular function and cardiovascular risk (Hill et al. 2003), severity of coronary artery disease (Thum et al. 2005), and reduced capillary density in patients with impaired glucose tolerance (Prior and Ryan 2013). The release of cytokines, chemokines, and growth‐promoting factors is hypothesized to be the major mechanism by which CAC influence endothelial growth and angiogenesis. However, few studies have evaluated the secretion of such factors from CAC in disease and physiological contexts. In this study, our major findings were as follows: (1) CFU‐CAC cultured in vitro from healthy men released the signaling proteins TGF*β*1, TNF*α*, and MCP‐1, (2) CAC secretion of TGF*β*1 was greater following acute exercise independent of glucose level, and (3) acute in vivo exercise failed to preserve in vitro NO release from CFU‐CAC in the hyperglycemic condition.

CFU‐CAC are a mixed population of cells including cells from the hematopoietic lineage such as T cells and monocytes (Rohde et al. 2006, 2007; Medina et al. 2010). CFU‐CAC cultures also display expression of endothelial‐related genes such as CD31, CD133, and KDR (Pula et al. 2009). CD4^+^ T‐cell stimulation via contact with CD14^+^ monocytes leads to paracrine release of T‐cell‐derived factors that are necessary for CFU colony formation (van Beem et al. 2008), indicating that different cell populations in CFU‐CAC work cooperatively to promote vascular effects. It has been suggested that CFU‐CAC represents the angiogenic potential of PBMC (Pula et al. 2009). CFU‐CAC have been shown to secrete angiogenic factors such as MMP‐9 and IL‐8 (Pula et al. 2009). In this study, we identified three cytokines; TGF*β*1, MCP‐1, and TNF*α* that were secreted from CFU‐CAC.

Transforming growth factor‐beta‐1 has pleiotropic effects on the vasculature and are likely context specific. In development, TGF*β* signaling is essential for proper vasculogenesis and TGF*β*1 localized to endothelial cells is required for angiogenesis (ten Dijke and Arthur 2007). In adult vessels, TGF*β*1 stimulates the release of other proangiogenic cytokines such as VEGF, and MCP‐1 (Vinals and Pouyssegur 2001; Ma et al. 2007). TGF*β*1 has anti‐inflammatory effects as TGF*β*1 decreases the expression of adhesion molecules on endothelial cells (Lebastchi et al. 2011) and activation of integrins in leukocytes leading to reduce endothelial transmigration (Basoni et al. 2005). In early stages of atherosclerosis, TGF*β*1 inhibits intimal smooth muscle cell accumulation, stimulates the extracellular matrix for repair, and controls inflammation whereas in advanced atherosclerosis, TGF*β*1 is related to excess vascular remodeling (Lutgens et al. 2002). Furthermore, upregulation of the TGF‐*β* pathway in retinal vessels is associated with proliferative diabetic retinopathy (Gerhardinger et al. 2009).

With regard to exercise, TGF*β*1 has been associated with a proangiogenic role in response to an acute bout of physical activity (Breen et al. 1996). Other studies highlight the role of exercise training to diminish the deleterious vascular remodeling effects of TGF*β*1. For example, physical deconditioning was related to higher skeletal muscle *Tgfb1* gene expression and conduit artery wall thickness, whereas 8 weeks of exercise training via functional electrical stimulation in spinal cord injury patients led to reduced skeletal muscle *Tgfb1* expression and decreased artery wall thickness (Lammers et al. 2013). Furthermore, aging‐associated TGF*β*1, collagen I, and collagen III expression in carotid arteries of mice were decreased by voluntary exercise (Fleenor et al. 2010). Therefore, the role of TGF*β*1 in exercise‐related adaptations is likely context specific for disease status. Our data revealed significant increases in TGF*β*1 protein from CAC exposed in vivo to a single bout of moderate treadmill exercise in both the normal and hyperglycemic in vitro culture conditions and increased *Tgfb1* gene expression in the high glucose condition with exercise. Inconsistency in the increase in TGF*β*1 gene and protein expression in the normal glucose condition with exercise may be related to differences in timing of the exposure, as exercise was performed acutely in vivo and glucose exposure occurred over 5 days of in vitro culture. Therefore, gene expression in response to acute exercise may have returned to baseline during the in vitro culture period in normal glucose conditions. Regardless, CAC are a potential source for TGF*β*1 in circulation with exercise. Interestingly, TGF*β*1 secretion was not associated with CFU‐CAC counts in this study. Therefore, TGF*β*1 does not appear to be necessary for colony formation, however, the angiogenic contribution of CAC‐derived TGF*β*1 should be evaluated further.

TNF*α* and MCP‐1 proteins were also detected in our CFU‐CAC culture supernatant and their secretion was significantly correlated with one another. This finding is consistent with the evidence showing that TNF*α* stimulates MCP‐1 expression in monocytes and endothelial cells in a diabetic environment (Yang et al. 2009). TNF*α* and MCP‐1 are inflammatory cytokines that have both detrimental and beneficial effects on the vasculature. TNF*α* is an inflammatory cytokine that induces endothelial cell activation and monocyte infiltration (Hopkins 2013). However, TNF*α* has also been shown to be important in the growth of collateral vessels (Arras et al. 1998). MCP‐1 is a C‐C family chemokine that recruits monocytes and is associated with atherosclerotic lesions (Hopkins 2013). On other hand, in a model of hindlimb ischemia, monocytes were recruited to the site of injury resulting in the production of MCP‐1 and stimulation of angiogenesis (Capoccia et al. 2008). Evidence suggests that exercise training reduces circulating TNF*α* and MCP‐1 levels in patients with cardiometabolic diseases (Adamopoulos et al. 2001; Troseid et al. 2004; Palmefors et al. 2014). Contrary to our hypothesis, neither acute exercise nor elevated glucose significantly changed the amounts of TNF*α* or MCP‐1 generated by CFU‐CAC suggesting that a short‐term exposures may not change the level of secretion of these cytokines. However, our results that TNF*α* and MCP‐1 are secreted form CFU‐CAC are corroborated by van Beem et al. (2008), who also reported the appearance of TNF*α* and MCP‐1 in the same culture system. They also reported that both cytokines were detected in media from activated T cells, although in lower concentrations than complete CFU‐CAC cultures suggesting that cells other than the T cells in culture produce TNF*α* and MCP‐1. Rohde et al. (2006) found that in vitro vascular networks were formed with supernatants from CFU‐CAC cultures and not supported when monocytes or T cells were depleted from the CFU‐CAC assay or when TNF*α* was quenched with antibodies, indicating an important role for TNF*α* in colonization. Therefore, inflammatory cytokines play a role in vessel regeneration and repair, however, a more complete understanding of their function in regenerative, exercise, and disease contexts is necessary.

Nitric oxide bioavailability is an important factor for vascular reactivity, vascular endothelial cell function, and is now recognized as significant for the mobilization and function of certain CAC types (Aicher et al. 2003; Jenkins et al. 2009). Diabetes is associated with reduced mobilization of CAC populations (Fadini et al. 2013) and CD34^+^ and CD34^+^/KDR^+^ CAC have been negatively related to fasting and postchallenge glucose levels (Fadini et al. 2007). CFU‐CAC number, CAC eNOS phosphorylation (Ser^1177^), and NO in CAC culture media was reduced when cells were incubated in 25 mmol/L glucose compared with 5 mmol/L (Chen et al. 2007). In our study, we did not observe a significant independent effect of glucose on NO secretion. These disparate results may be due to our choice of 15 mmol/L for the high glucose condition – a glucose concentration that is more similar to that which may be observed in humans with uncontrolled diabetes.

We observed that NO in CAC conditioned media was not maintained with acute exercise under high glucose conditions. Habitual physical activity, acute exercise, and reduced physical activity have effects on CAC NOi. Jenkins et al. (2009) reported that CFU‐CAC eNOS gene expression and NOi was higher in active compared with inactive young men at rest, but NOi was only higher in CFU‐CAC from inactive men after acute exercise. CFU‐CAC NOi has been shown to also be lower after 10 days of reduced physical activity in active men (Guhanarayan et al. 2014). While NOi appears to be important for CAC function, and is influenced by physical activity, acute exercise preconditioning could not abrogate the effects of glucose on NO secretion in our study. It is possible that repeated bouts of acute exercise or exercise training may have a greater impact on preserving NO secretion upon exposure to high levels of glucose. Oxidative stress compromises NO bioavailability in CAC from diabetic patients (Jarajapu et al. 2011), and oxidative stress may be altered in CAC with exercise (Jenkins et al. 2009, 2011) therefore future investigations should evaluate the interaction between exercise, oxidative stress, and diabetic conditions on the regulation of NO in CAC.

Finally, our study is not without limitations. First, since CFU‐CAC are comprised of a mixed population of cells, it remains unclear which cells in culture are responsible for the secretion of the investigated factors. However, we believe that in vivo cell types contained in the CFU‐CAC culture system work in concert and may be necessary to confer the relationship with CVD and endothelial function. Additionally, the soluble factors we evaluated herein were chosen a priori and it is likely that there are multiple other factors that may be influenced by changes in glucose concentration and exercise. A proteomics or cytokine array approach could greatly improve our understanding of the complement of soluble factors released by CAC populations under various conditions. The participants in our study were young, healthy active men. It would be informative to evaluate the effect of exercise on CFU‐CAC cytokine secretion in patients with insulin resistance or diabetes. Lastly, conditions associated with diabetes other than glucose (such as insulin and insulin resistance) may impact CAC cytokine secretion therefore future studies should evaluate other diabetes‐related environments.

## Conclusion

Evaluation of the secretome of cells associated with vascular health and function may provide important information about cardiometabolic disease. Our data contribute novel evidence that one of the major ways CAC contribute to vascular health and repair, cytokine secretion, can be influenced by exercise and conditions simulating disease. Understanding how circulating cells promote vascular repair through soluble factor secretion has the potential to inform regenerative therapies for disease.

## Conflict of Interest

The authors have no conflicts of interest to report.
